# Supplementary material for: The interactome of intact mitochondria by cross-linking mass spectrometry provides evidence for coexisting respiratory supercomplexes
Source: Mol Cell Proteomics. 2017 Dec 8;17(2):216–32. doi: 10.1074/mcp.RA117.000470 (PMC5795388; doi:10.1074/mcp.RA117.000470)
Supplement: Supplemental Data [file supp_RA117.000470_133922_0_supp_23978_fzffwf.zip › spectra_annotation/new_mito_protein_spectra_annotation/274-1-385-1-797-1.pdf]

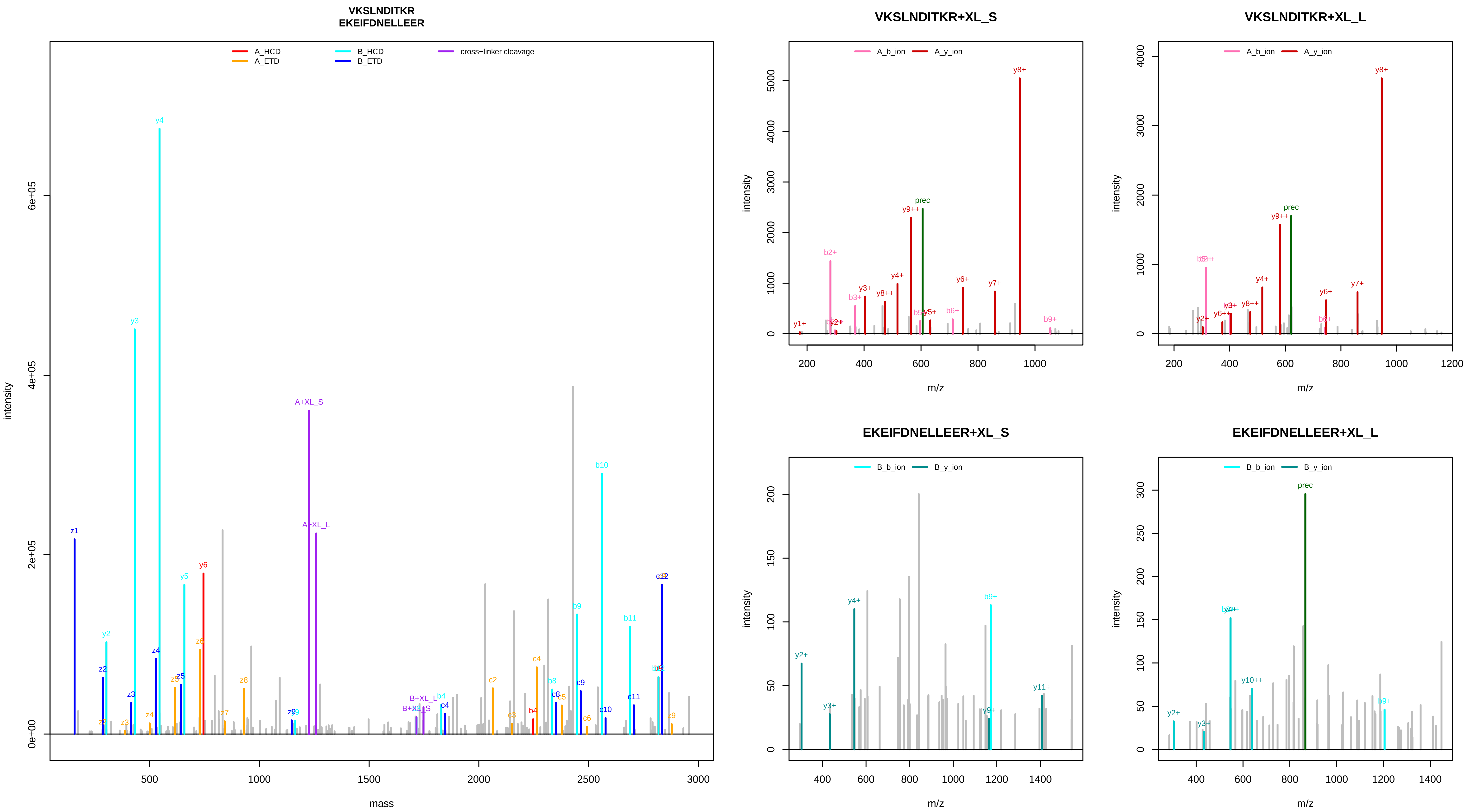

VKSLNDITKR
EKEIFDNELLEER
A_HCD
A_ETD
B_HCD
B_ETD
cross−linker cleavage
intensity
mass
0e+00
2e+05
4e+05
6e+05
500
1000
1500
2000
2500
3000
z1
y2
z2
y3
z3
y4
z4
y5
z5
y6
z6
z8
A+XL_S
A+XL_L
B+XL_L
B+XL_S
b4
c4
c2
c3
b9
b8
c8
c9
b10
c10
b11
c11
c12
VKSLNDITKR+XL_S
VKSLNDITKR+XL_L
A_b_ion
A_y_ion
m/z
y8+
prec
y9++
b2+
y4+
y3+
y8++
y6+
y7+
b3+
y1+
b6+
b9+
EKEIFDNELLEER+XL_S
EKEIFDNELLEER+XL_L
B_b_ion
B_y_ion
y2+
y4+
y3+
b9+
y11+
y9+
y10++
